# Arthrofibrosis Following Anterior Cruciate Ligament Reconstruction

**DOI:** 10.5704/MOJ.1911.006

**Published:** 2019-11

**Authors:** I Rushdi, S Sharifudin, A Shukur

**Affiliations:** Department of Orthopaedics, Hospital Teluk Intan, Teluk Intan, Malaysia

**Keywords:** anterior cruciate ligament reconstruction, complications, arthrofibrosis, pre-operative patient selection, postoperative rehabilitation

## Abstract

**Introduction:** Anterior cruciate ligament (ACL) tear is a frequent injury and its reconstruction is among the most commonly performed orthopaedic surgical procedures. ACL reconstruction generally yields good results. However, its recovery can be hampered by the development of postoperative complications. The aim of this study was to review complications following arthroscopic ACL reconstruction done in Hospital Raja Permaisuri Bainun, Ipoh and Hospital Teluk Intan, Perak with the emphasis on arthrofibrosis. Arthrofibrosis is defined as a loss of 15 degrees extension or more, with or without flexion loss compared to the contralateral knee.

**Materials and Methods:** The study is based on a series of retrospective cases, on which medical records of 200 patients who underwent ACL reconstruction surgeries between March 2007 and December 2014 were reviewed. Follow-up treatment records were available for 166 patients (83%). The data was analysed to identify the risk factors for developing complications with an emphasis on arthrofibrosis.

**Results:** Eight patients (5%) developed arthrofibrosis in the post-operative period. Early surgical intervention, preoperative limited range of motion and female gender are the risk factors correlate with arthrofibrosis. However, the type of graft used and meniscal procedure do not have a significant correlation with the development of arthrofibrosis. Other complications encountered are local infections, hypertrophic scar and chronic regional pain syndromes.

**Conclusion:** The 5% incidence of arthrofibrosis following an ACL reconstruction in our centres can be reduced with proper preventive measures which include thorough preoperative evaluation, proper patient selection, restoration of ROM prior to surgery and proper timing of surgery.

## Introduction

The anterior cruciate ligament (ACL) is the most frequent ligament to be injured in the knee joint^1, 2^, accounting for about 50% of all ligament injuries, and has an incidence of about 30 per 100,000 heads of population per year^3^. ACL reconstruction surgery is among the most frequent performed orthopaedic procedure and generally yields good results but patient recovery can be hampered by the development of post-operative complications^4, 5^.

General complications from ACL reconstruction surgery are wound problems, effusion, infections^6^ and venous thrombosis^7^. Complications specific to the patella tendon autograft are patella fracture and patella tendon rupture. Other complications are arthrofibrosis, patellofemoral pain, local tenderness^8^ and loss of sensitivity^4^.

There is no universally accepted definition of arthrofibrosis. Historically, arthrofibrosis can be defined as a loss of motion after a knee trauma or reconstructive surgery^9, 10, 11^. Based on literatures, its overall prevalence is about 4-38%^12^. Shelbourne and associates classified arthrofibrosis based on the pattern and severity of knee stiffness^13^ (Table I). Type 3 and 4 arthrofibrosis are indicated for surgical intervention.

**Table I:** Classification of arthrofibrosis

TYPE	EXTENSION	FLEXION	PATELLA MOBILITY
Type 1	<10° extension loss	Normal flexion	Normal
Type 2	>10° extension loss	Normal flexion	Normal
Type 3	>10° extension loss	>25° flexion loss	Decreases
Type 4	>10° extension loss	>30° flexion loss	Decrease and patella infera

Factors contribute to arthrofibrosis are genetic predisposition of the patient himself, types of injury such as multiple ligaments injury, dislocations, associated infection and synovitis. Surgical factors contributing to arthrofibrosis are timing of surgery, preoperative range of motion, malposition of graft, excessive graft tension, associated extra-articular procedure and meniscal repair. Post-operative factors such as prolonged immobilisation, poor rehabilitation, complicated with reflex sympathetic dystrophy, infections and synovitis can also contribute to arthrofibrosis^14^.

The main objective of our study was to review complications following arthroscopic ACL reconstruction, done in Hospital Raja Permaisuri Bainun, Ipoh and Hospital Teluk Intan, Perak with the emphasis on arthrofibrosis.

## Materials and Methods

Our study was an observational study of case series. Ethical clearance was obtained before reviewing patient records who underwent ACL reconstruction performed by the same surgeon from March 2007 till December 2014 in Hospital Raja Permaisuri Bainun, Ipoh and Hospital Teluk Intan, Perak, Malaysia.

Data compiled were demographic information, mechanism of injury, other associated injuries, timing of surgery, preoperative range of motion, graft chosen, post-operative range of motion, post-operative complications and other related procedures.

The collected data was then screened and filtered according to inclusion and exclusion criteria. The studied patients must fulfil several pre-operative selection criteria and were compliant to post-operative rehabilitation programmes. Exclusion criteria were patients who defaulted follow-up, had multi-ligamentous injuries and those who were complicated with post-operative infection or chronic regional pain syndrome (CRPS).

Data was reviewed and analysed to identify the risk factors for developing arthrofibrosis. All surgeries were performed on patients who were under spinal or general anaesthesia. The ACL reconstruction was performed arthroscopically with autograft taken from hamstring or bone patella tendon bone graft. Hamstring graft was taken from ipsilateral semitendinosus and gracilis tendon prepared into two-strand graft or three-strand graft preparation depending on the length and diameter. Each end of graft was stitched together with a high strength of non-absorbable suture. All graft fixations used bio absorbable screws except the femoral fixations for the hamstrings grafts which were loaded with extracortical buttons. In addition, the graft tunnel contact was preserved at least 20mm in length.

Rehabilitation started immediately post-operative where the patient’s knee was put on knee brace locked in full extension. The knee was kept in full extension with the help of a pillow under the ankle. On day one post-operation, five exercises were taught to patients which include ankle pump exercise, passive quadriceps strengthening, straight leg raised, patella mobility and knee active-assisted ROM exercise. The knee was kept in full extension except during ROM exercises. The patient with no concomitant meniscal repair procedure was allowed to weight bear while the patient who had the meniscal repair procedure was instructed not to weight bear on the affected limb for six weeks. Strict instructions were given to our patients on early ROM exercise with the help of physiotherapy sessions. For patients with bone patella tendon bone graft, a rehabilitation protocol was adapted from the accelerated rehab protocol by Shelbone KD^18^ while patients with hamstring graft had to follow conventional rehabilitation programs^10^.

Post-operative follow-ups were scheduled starting from week 2, week 6, week 12 and after 6 months, post-operation. The range of motion was recorded at each interval. Our targeted ROM was 30° incremental every two weeks for both types of graft.

We classified a patient to have arthrofibrosis if there was an extension limitation of 10° or more with or without flexion and patella mobility limitation. At week six follow-up, if the targeted ROM of 90° flexion and < 5° extension lag were not observed, the patient would be subjected to aggressive physiotherapy sessions. If there was still no improvement at week 12, the patient would be subjected to arthrolysis.

## Results

There were 200 recorded patients, on which 142 patients were from Hospital Raja Permaisuri Bainun and 58 patients from Hospital Teluk Intan. A total of 166 patients (83%) completed their follow ups. The mean age of our patients was 26 years, of which 154 (93%) were males. The most common cause of injury was sports injury (Table II). Arthrofibrosis was observed in eight out of 166 patients (4.8%) at week six of post-operative. At the time of surgery, three patients had pre-existing limitation motion of flexion (90°-110°) and extension lag (5°-15). All the three patients who went to surgery with a limited motion were complicated with arthrofibrosis. There were 12 female patients who had underwent ACL reconstruction surgeries. Two of them developed arthrofibrosis during week six post-operatively. The average time of surgery was at 3.4 months (3 months and 2 weeks) post injury with one patient underwent ACL reconstruction at two weeks post injury. A total of 110 patients in this study used hamstring graft while the remaining 56 patients used bone patella tendon bone (BPTB) graft. Five patients in the hamstring group (5%) and three in the BPTB group (5%) developed arthrofibrosis. One hundred five patients also underwent meniscal procedures apart from ACL reconstruction. Four of them developed arthrofibrosis (3.8%).

**Table II:** Demographic data

Age (years, mean, range)	26 (18-45)
Sex	
Male	93% (154)
Female	7% (12)
Race	
Malay	82%
Indian	10%
Chinese	7%
Others	1%
Causes of injury	
Sport activity	73%

Patients who developed arthrofibrosis had to undergo aggressive physiotherapy sessions once arthrofibrosis was detected. Five of them were subjected to arthroscopic adhesiolysis and all restricted knee movements were resolved.

## Discussion

In our study, three factors were found to be correlated with arthrofibrosis, namely pre-operative limited motion, the time of surgery and female patients. Three out of eight patients (37.5%) who developed arthrofibrosis went to surgery with pre-existing limited range of flexion motion (90°-110°) and extension lag (5°-15). Only these three patients were subjected to ACL reconstruction while their motions were limited and all of them had developed arthrofibrosis postoperatively (100%).

Our findings on the correlation between pre-operative range of motion and arthrofibrosis development were also observed by Quelard *et al*^16^. In their study, they found that limited pre-operative range of motion among other factors was found to be significantly correlated with delayed recovery. Mayr *et al*^14^ and Cosgarea *et al*^17^ observed the same correlation between limited pre-operative knee motion and stiffness after ACL reconstruction. Motion deficit was caused by high inflammatory changes in an acutely injured knee. They suggested that risk of arthrofibrosis was higher in cases of early ACL reconstruction of inflamed knee that usually presented with limited range of motion. Johnson *et al*^20^ also demonstrated that ROM recovery was slower when the ruptured ACL was associated with bone contusions which significantly related to limited post-operatively ROM.

The time of surgery also correlated to higher risk of arthrofibrosis as the only one patient who went to surgery at two weeks post injury developed arthrofibrosis while other cases which were done at average 3.4 months post injury has lower risk of developing arthrofibrosis.

Shelbourne *et al*^10^ stated that arthrofibrosis as a potential complication of acute ACL reconstruction. They did a retrospective study on 169 acute ACL reconstructions in a population of young athletes to determine the optimal time to perform acute ACL reconstruction with respect to arthrofibrosis. They found that delaying reconstructive surgery at least three weeks from the time of injury would result in earlier return of strength and more importantly, a significant decrease of arthrofibrosis incidence. The time of surgery as a correlating factor was also revealed by Quelard *et al*^16^ where they observed a high risk of difficult rehabilitation for patients undergoing surgery within 45 days of injury without having achieved full knee ROM (80% delayed recovery). Wasilewski *et al*^15^ had shared a similar finding, where they found the majority of arthrofibrosis occurred when surgeries were done on an acute knee and post-operatively motion recovery was significantly less for the ACL reconstruction in an acute phase.

In our study, two out of 12 female patients developed arthrofibrosis putting female patients who underwent ACL reconstruction to face higher risk of developing arthrofibrosis at 16%, higher than male patient which is at 4%. Our finding on the correlation between female patients with the risk of developing arthrofibrosis was also shown by Quelard *et al*^16^ but they could not explain the reason of the correlation. However, this correlation was opposed by Cosgarea *et al*^17^.

We also observed two other factors that were not correlated with the development of arthrofibrosis, namely type of graft used and concomitant meniscal procedure. Five out of 110 patients of the hamstring group (4.5%) developed arthrofibrosis similar to BPTB group where three out of 56 patients (5.3%) had similar complications. Four out of eight patients (50%) who developed arthrofibrosis also had concomitant meniscal procedure showing both groups had the same risks of developing the complications.

Splinder *et al*^19^ had similar findings on the type of graft choice. In his systematic review, he identified nine randomised controlled trials comparing many factors on successful outcomes of anterior cruciate ligament reconstruction. He found that graft type may not be the primary determinant for successful outcomes after an anterior cruciate ligament surgery. Cosgarea *et al*^17^ who studied the effects of concomitant meniscal procedure on the risk of developing arthrofibrosis post ACL reconstruction, has shared the same findings.

There are also studies on rehabilitation impact on arthrofibrosis post ACL reconstruction. Shelbourne *et al*^18^ studied the effects of an accelerated versus conventional rehabilitation program, while Noyes *et al*^11^ studied the effects of early range of motion. They found that early motion eliminated the significant risk of arthrofibrosis post ACL reconstruction.

## Conclusion

Our seven years records of follow-up on arthrofibrosis post ACL reconstruction show that, factors that can be correlated with arthrofibrosis are pre-operative limited motion, the time of surgery and female patients. However, further study is needed to determine the association of these factors with arthrofibrosis.

With this limited observation, the incidence of arthrofibrosis following an ACL reconstruction can be further reduced with proper preventive measures which include thorough preoperative evaluation, proper patient selection, restoration of ROM prior to surgery and proper timing of surgery.
